# Supplementary material for: Learning curve and surgical outcome of robotic assisted colorectal surgery with ERAS program
Source: Sci Rep. 2022 Nov 29;12:20566. doi: 10.1038/s41598-022-24665-w (PMC9709162; doi:10.1038/s41598-022-24665-w)
Supplement: Supplementary file 1 — Supplementary Information 1. [file 41598_2022_24665_MOESM1_ESM.zip › LSC Raw data-Ñ[▒K/050/HS_ñw╝╨░O▒Kñσ.pdf]

正式摘要(電子簽章：000000178329525)

W95 / W95 ☐英文標題(注意:無英文姓名-請補病人基本檔!) ☐【讀取】憑證

# 臺中榮民總醫院

## 出院病歷摘要

轉入號： 轉出號：

[redacted]

| | |
|---|---|
| (9)入院日期 | 108/10/14 (2019) 直腸外科 W95 - 37 |
| (10)轉科日期 | |
| (11)出院日期 | 108/10/23 (2019) 住院天數計 9 日 |

### 診斷

- 入院 :Rectum cancer, s/p CCRT
- 出院 :Rectum cancer, s/p CCRT, s/p laparoscopic LAR ISR+RT LLND+ ileostomy on 2019/10/15, pTxN0(0/7, 0/2)M0

### 主訴

- Rectum cancer s/p CCRT, for surgery

### 病史

- This 63 y/o female has the underlying disease of hypertension.
  She visited Dr. Lin's OPD with the symptoms of intermittent anal bleeding since elarlier this year. Colonoscopy in June, showed Rectal tumor 2cm FAV, and biopsy report showed Adenocarcinoma. Lab data showed elevated CEA level 6.62ng/mL, CA199 within normal range. Complete tumor survey was done. Whole body PET/CT showed C/W rectal cancer, with no definite evidence of FDG-avid tumor elsewhere, MRI of pelvis showed C/W rectal cancer, and no obvious lymphadenopathy noted over bilateral iliac region. Chest CT showed no abnormal lung nodule. CCRT first was sguuested, and the patient finished the treatment course. The follow-up colonoscopy exam showed regression rectum tumor 2 cm FAV, and decreased CEA level 1.72 ng/mL.
  She was admitted for scheduled surgery and further management.

### 體檢發現

- Anoscopy: mass on 6"

  Evaluation

  PE:

  Tumor marker CEA CA-199
  2019/6 6.62
  2019/8 1.7

  image abd CT MRI
  2019/6 - Rt pelvis int. iliac LN 0.4cm.
  mesorectum LN*4
  colonsocopy
  2019

  SE
  2018

  Dx:
  1081002 No side effect

  - General appearance :
    - easy looking , conscious clear , without cardiopulmonary distress
    - Vital signs自動帶入date 體溫(BT) 脈搏(P) 呼吸(R) 血壓(BP) 身高(BH) 體重(BW) BMI
      20191014 36.5℃ 59-次/分 18-次/分 137/72mmHg 150.8cm 58.4kg 25.7
    - Vital signs: B.P. 137/72 mmHg , P.R. 59 /min , R.R. 18 /min , B.T. 36.5 ℃
  - HEENT :
    - Eyes: no anemic conjunctiva, no icteric sclera, normal EOM
    - ENT: no otorrhea, no rhinorrhea, no erythematous mucosa, no injected tonsil, no deformity
  - Neck: supple, freely movable, trachea midline,no deformity, no engorged jugular vein , no palpable thyroid or lymph node enlargement
  - Chest:
    - Inspection: Grossly normal, symmetric and fully expansion, no accessory muscle used, no operation scar, no spider angiomata, no gynecomastia
    - Palpation: no palpable mass or nodule, no subcutaneous emphysema, no tenderness
    - Ausculation: clear breathing sound
  - Heart:
    - Palpation: no thrill, no heave
    - Percussion: normal heart size
    - Ausculation: regular heart beat, normal S1, S2, no gallop, no murmur
  - Extremities: No deformity, full ranged of motion without limitation, no clubbing finger or nail cyanosis, no pitting edema, no ulcer or pigmentation, no obvious skin lesion, no varicose vein, no bone, joint or muscle tenderness

  **CRS CONDITION**

  - Karnofsky performance status: (100) Normal, no complaints, no evidence of disease
  - PE of Abdomen:
  - __1. Inspection: flat
  - CEA: 1.72 ng/ml
  - Imaging Studies:
  - Colonoscope report: Rectum ca 2 cm FAV s/p CCRT
  - Sono , CT of abdomen and chest Films:檢驗項目 :CT ABDOMEN-SURVEY 報告日期 :1081001
    主 訴 :
    Result:
    Finding:
    Post IV contrast CT scan of the whole abdomen with oral
    contrast showed:
    a case of lower rectal ca. preoperation survey.
    Atrophy of right kidney with mild hydronephrosis and renal stone about 1.2 cm. Left several small
    renal cysts.
    GB stone about 1 cm.
    Multiple low density at both lobes of liver less than 4.1 cm. Stable as compared with previous CT
    2019/7/10 chest CT. Favored cyst.
    Multiple small paraaortic LNs.
    No other specific findings noted at spleen, pancreas.
    DJD change noted at lower T and L spine.
    Impression:
    No evident distal metastasis in this study.
    For other details, please see the above descriptions.

- 檢驗項目 :CT SCAN,CHEST, HILA, TRACHEA 報告日期 :1080729
  主 訴 :
  Result:
  Finding:
  Contrast enhanced CT scan of chest, shows:
  1. Rectum ca for tumor survey.
  2. Calcified node in mediastinal node and right hilar node.
  3. No abnormal lung nodule.
  4. LVH. Dilated LA.
  5. Multiple hepatic cysts.
  6. Gallstone.
  7. Hyplasia of right kidney. Hypertrophic change of left kidney. Left renal cyst.
  8. Otherwise, no other significant finding.
  Impression:
  Rectum ca for tumor survey.
  No abnormal lung nodule.
  Calcified node in mediastinal node and right hilar node.
  檢驗項目 :MRI - PELVIS 報告日期 :1080702
  主 訴 :
  Result:
  Finding:
  Imaging Report Form for Colorectal Carcinoma (AJCC 8th)
  1. Imaging Date: 20190701
  Imaging Modality, Imaging by
  MRI
  Imaging Protocol:
  MRI of pelvis without and with iv constrast
  2. Tumor location / Size
  Location:
  Rectum
  Size:
  Non-measurable
  3. Tumor invasion
  Tumor within colonic or rectal wall
  4. Regional nodal metastasis
  Not assessable
  5. Distant metastasis (In this study)
  No or Equivocal
  6. Other findings:
  1. Asymmetrical wall thickening of rectum with perirectal fat stranding, compatible with rectal cancer. Please correlate with colonoscopy.
  2. No obvious enlarged lymphadenopathy over bilateral iliac region is noted.
  3. Mass lesions up to 4.6cm in uterus, in favor of myomas.
  4. UB wall thickening, R/O chronic cystitis.
  5. Smaller than 1.0cm ovarian cysts are also noted.
  Impression:
  Rectal cancer
- Figures:
  1. colon & rectum

## 手術日期及方法

- 手術日期及方法20191016 Low anterior resection with colo-anal anastomosis Lymphadenectomy, pelvic Ileostomy Technical fee of 3D complex minimally invasive surgery≧5 hrs

## 住院治療經過

- After admission, pre-OP assessment was done, and the lab data showed no siginificant abnormality needed to be corrected before the surgery. TaTME+ LLND was performed smoothly on 2019/10/23. After the operation, she was sent back to general ward for post-OP care. Foley was removed on 10/17. The rod of the ileostomy was removed on 10/22. Her drainage was clear, so the drainage tube was removed on 10/23. She is now under semi-liquid diet, and there was no discomfort. Under stable condition, she was discharged on 2019/10/23.

## 合併症

## 一般檢查

- 累積報告 :

| DATE | TIME | ALB | TP | BIL.T | BIL.D | ALKP | AST | ALT | LDH | CHOL | TG | HDL-C | LDL | UA | NH3 | AMY |
|---|---|---|---|---|---|---|---|---|---|---|---|---|---|---|---|---|
| 1081014 | 1643 | 4.1 | | 0.84 | 0.23 | 84 | 71 | 72 | 201 | | | | | | | |
| 1081016 | 0919 | 3.5 | | 0.6 | 0.2 | | 42 | 45 | | | | | | | | |
| 1081021 | 0910 | 3.1 | | 0.54 | | | | 21 | | | | | | | | |

| DATE | TIME | WBC | RBC | HGB | HCT | MCV | PLT | BLST | NEUT | BAND | SEG | LYM | MON | EOS | BAS |
|---|---|---|---|---|---|---|---|---|---|---|---|---|---|---|---|
| 1081014 | 1610 | 3530 | 3.95 | 12.0 | 35.3 | 89.4 | 105 | | 76.3 | | | 12.7 | 8.2 | 2.5 | 0.3 |
| 1081016 | 0419 | 3750 | | 11.1 | | | 109 | | 87.4 | | | 8.3 | 4.0 | 0.3 | 0.0 |
| 1081021 | 0957 | 4820 | 3.77 | 11.5 | | | 174 | | 76.2 | | | 8.3 | 12.2 | 2.7 | 0.6 |

| DATE | TIME | NA | K | CL | CA | BUN | CREAT | Mg | P | CRP | ESR | PCT | LIPASE | LACTATE |
|---|---|---|---|---|---|---|---|---|---|---|---|---|---|---|
| 1081014 | 1643 | 140 | 3.9 | 104 | 8.9 | 16 | 1.07 | | | | | | | |
| 1081016 | 0919 | 140 | 3.5 | | | 17 | 1.11 | | | | | | | |
| 1081021 | 0910 | 141 | 4.3 | | 8.3 | 8 | 0.92 | | | | | | | |

| DATE | TIME | GLUCOSE | KETONE | CK | CKMB | PT-P | PT-C | APTT-P | APTT-C | TRO-T |
|---|---|---|---|---|---|---|---|---|---|---|
| 1081014 | 1610 | 94 | | | | | | | | |
| 1081014 | 1611 | | | | | 9.8 | 10.1 | 29.4 | | |

## 特殊檢查

## 病理報告

- 活體組織切片病理報告 手術日期：**108/10/16** 病理號：**S0836617**

  診斷醫師:[illegible] 報告日期:108/10/22
  Pathologic diagnosis:
  1. Rectum, TaTME --- a. Status post CCRT with no residual tumor. The bilateral cut ends and mesocolic lymph nodes (0/7) are free of tumor.
  b. Hyperplastic polyps.
  2. IMA root, dissection --- Unremarkable.
  3. Lymph node, right pelvic, dissection --- Unremarkable (0/2).
  Prognostic and predictive factor:
  1. Tumor size: Cannot be assessed.
  2. Depth of invasion: No evidence of primary tumor.
  3. Histologic grade: GX: Cannot be assessed.
  4. Angiolymphatic invasion: Not identified.
  5. Perineural invasion: Not identified.
  6. Tumor deposits (discontinuous extramural extension): Not identified.
  7. Surgical Margins: a) Proximal Margin: Uninvolved, distance of stitch from margin: 14 cm. b) Distal Margin: Uninvolved, distance of stitch from margin: 1 cm. c) Radial or Mesenteric Margin: Uninvolved by invasive carcinoma, distance of stitch from margin: 15 cm.
  8. Lymph node status: Uninvolved (0/7).
  9. Extranodal involvement: Not applicable.
  10. Tumor regression grading S/P CCRT: Present, no viable cancer

- cells (complete response, score 0).
  11. Type of polyp in which invasive carcinoma arose: Not applicable.
  12. Additional pathologic findings: Hyperplastic polyp.
  13. Pathological TNM Stage: ypTxN0(According to the eighth edition, 2017, American Joint Committee on Cancer Staging Guidelines for Tumors / 2017 CAP guideline).
  14. TNM descriptors: y (Post-treatment).
  Gross description:
  The specimen consists of 1) a segment of rectum, 15 cm in length and 5 cm in proximal cut end circumference, 6 cm in distal circumference, and perirectal soft tissue. A stitch is noted 1 cm above the nearest cut end. No tumor is seen grossly on cut. The lymph node is hard to find grossly. 2) a piece of tan gray soft tissue, 3.5x2x1 cm, labeled as "IMA root". 3) a piece of tan gray soft tissue, 6.5x3x0.4 cm, labeled as "right pelvic lymph node". All for section: A) specimen 2; Representative part for section: B) specimen 3 C) proximal cut end D) distal cut end E) cut end F-J) mucosa near the stitch K) polyps L-V) lymph node.
  #T-59600_2 #M-09183_2 0 1105 00SP00
  #T-C4520_2 #M-09183_2 0 1105 000000

## 其它

## 出院時情況

- 3.改門診治療

## 出院指示

| 藥名 | 劑量 | 劑量單位 | 用法 | 頻次 | 數量 | 數量單位 | 現況 | 天數 | 單位 |
|---|---|---|---|---|---|---|---|---|---|
| Esomeprazole tab | 1 | TAB | PO | QDAC | 7 | TAB | TH | 7 | 天 |

## 出院計劃

- 下次預約掛號資訊
  1081030 (P_下午) 大腸直腸 2448診 25號

主治醫師_2222H 林俊余_______________ / 住院醫師_2347D 許菀寧_______________

正式摘要(電子簽章：**000000178329525)**

W95 / W95

doctorRank:B
